# Case report: Myocarditis with nonsustained ventricular tachycardia following COVID-19 mRNA vaccination in a female adolescent

**DOI:** 10.3389/fped.2022.995167

**Published:** 2022-11-21

**Authors:** Jeongho Han, Joowon Lee, Sujin Choi, Hyunju Lee, Young Hwan Song

**Affiliations:** ^1^ Department of Pediatrics, Seoul National University Children's Hospital, Seoul, South Korea; ^2^ Department of Pediatrics, Seoul National University Bundang Hospital, Seongnam, South Korea; ^3^ Department of Pediatrics, Seoul National University College of Medicine, Seoul, South Korea

**Keywords:** adolescent, myocarditis, COVID-19, vaccine, case report

## Abstract

Children with underlying medical conditions potentially develop severe illness from Coronavirus disease 2019 (COVID-19). The use of vaccines against COVID-19 is currently recommended for the pediatric population. The COVID-19 vaccine has a temporal association with the occurrence of myocarditis. Although most patients with COVID-19 vaccination-associated myocarditis (C-VAM) exhibit a mild clinical course and rapid recovery, C-VAM potentially causes electrical instability and sudden cardiac death. Herein, we report the case of a 17-year-old woman who presented with chest pain and syncope following the first dose of the messenger RNA COVID-19 vaccine. The patient's heart function was impaired, and nonsustained ventricular tachycardia was frequent. Cardiac magnetic resonance (CMR) imaging satisfied the criteria for myocarditis. Despite the administration of immunomodulatory drugs, the patient's heart function was not fully restored, and the concentration of cardiac enzymes remained above the normal range. Persistence of late gadolinium enhancement was observed on short-term follow-up CMR imaging. Although most patients with C-VAM exhibit mild symptoms, significant cardiac arrhythmias potentially occur. Furthermore, some patients with C-VAM demonstrate prolonged impaired heart function and sustained late gadolinium enhancement on follow-up CMR imaging. Therefore, monitoring of electrical and functional cardiac abnormalities in patients with C-VAM is crucial and the long-term outcomes and prognosis of patients with C-VAM require further investigation.

## Introduction

Coronavirus disease 2019 (COVID-19) is a worldwide health problem, as it has reached pandemic level and caused multiple outbreaks globally. Although COVID-19 infection in children is typically asymptomatic or mild, it potentially progresses to severe illness in children with underlying medical conditions (1). Multisystem inflammatory syndrome in children, which is associated with high morbidity and mortality, can develop after COVID-19 infection (2). The use of vaccines against COVID-19 has increased among children and adolescents. Both the Pfizer-BioNTech BNT162b2 and Moderna mRNA-1273 vaccines have exhibited excellent efficacy and safety in the pediatric population (3, 4). The Centers for Disease Control and Prevention (CDC) recommended the use of the vaccine for adolescents aged ≥12 years on May 12, 2021, and for children aged 5–11 years on November 2, 2021 (5). The Korea Advisory Committee on Immunization Practices also recommended extending the use of the vaccine to persons aged ≥12 years on August 25, 2021.

Myocarditis and pericarditis are complications that potentially occur after COVID-19 vaccination (6–16). In most cases of COVID-19 vaccination-associated myocarditis (C-VAM) after BNT162b2 or mRNA-1273 vaccination, symptoms developed within a few days after the second vaccine dose, and the clinical course usually appeared mild with resolution of symptoms and signs within 1 week. However, fulminant myocarditis and sudden death after vaccination have also been reported (17, 18).

Herein, we describe the case of a 17-year-old woman with myocarditis after the first dose of the BNT162b2 vaccine. This patient, who presented with syncope and experienced several episodes of polymorphic nonsustained ventricular tachycardia, exhibited a chronic clinical course, which is an uncommon finding in other patients with C-VAM.

## Case description

A previously healthy 17-year-old woman presented with syncope 7 days after her first BNT162b vaccine dose. The patient was obese, with a body mass index of 26.6 kg/m^2^ (above the 95 percentile for their age and sex). Two days after the vaccination, the patient started experiencing generalized malaise, headache, chest pain, and dyspnea on exertion. Three days later, the patient experienced palpitations, and the intensity of her chest pain increased. The patient visited the emergency department of our hospital. The patient's electrocardiogram exhibited low voltage in the limb leads, and her troponin I concentration was within the normal range (0.032 ng/ml; normal: 0–0.045 ng/ml). The patient's symptoms were improved slightly, resulting in her subsequent discharge. The next day, the patient lost consciousness for several minutes while sitting in a restaurant.

The patient's vital signs on arrival were as follows: blood pressure, 102/60 mmHg; heart rate, 92 beats/min; respiratory rate, 19 breaths/min; temperature, 37.7 °C; and oxygen saturation, 100%. Physical examination revealed no remarkable findings. Neither audible murmurs nor signs of congestion were observed. Laboratory blood tests revealed myocardial injury without systemic inflammation. The level of high-sensitivity cardiac troponin I was 0.072 ng/ml (normal: 0–0.045 ng/ml). The patient's white blood cell count was elevated (10,960/uL; normal: 4,000–10,000/ul), and eosinophilia was absent. The C-reactive protein concentration (<0.40 mg/dl; normal: 0–0.5 mg/dl) and erythrocyte sedimentation rate (17 mm/h; normal: 0–20 mm/h) were not elevated. The patient's nasopharyngeal polymerase chain reaction (PCR) test result was negative for COVID-19. PCR tests for other viruses using nasopharyngeal swabs, blood, and stool samples were negative. Although serum neutralizing antibody titers against adenovirus types 2 and 5 were 1:180 and 1:256, respectively, nasopharyngeal PCR tests were negative for adenovirus.

**Figure 1:**
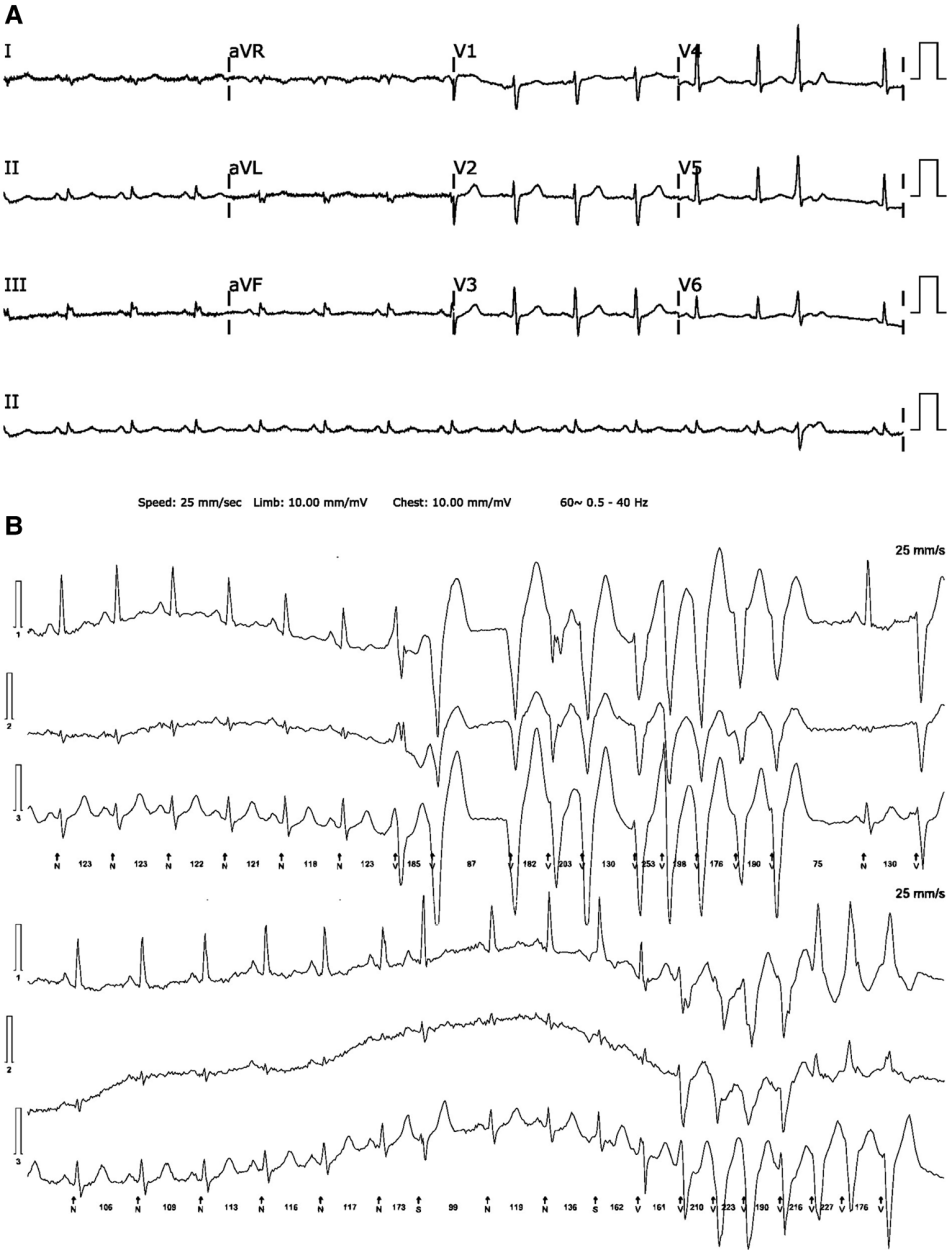
(**A**) A 12-lead electrocardiogram reveals low QRS voltage in limb leads and isolated ventricular premature beat. (**B**) Ambulatory 24-hour Holter monitoring reveals several runs of nonsustained polymorphic ventricular tachycardia.

**Figure 2:**
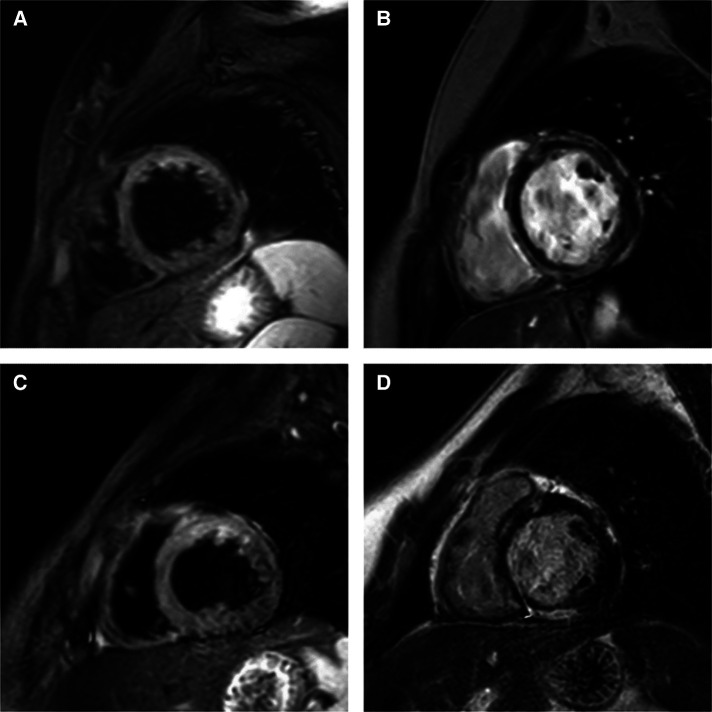
Short-axis cardiac magnetic resonance imaging on days 2 (**A,B**) and 18 (**C,D**) after the patient's presentation. (**A**) High signal intensity in anterior wall of left ventricle on T2-weighted image. (**B**) Diffuse multifocal patchy late gadolinium enhancement (LGE). (**C**) Sustained high signal intensity in anterior wall of left ventricle on T2-weighted image. (**D**) Persistent multifocal patchy LGE.

Electrocardiography revealed low voltage in the limb leads and premature ventricular contraction (Figure 1). There was no abnormal finding on the chest radiograph. Transthoracic echocardiography revealed left ventricular dilatation with a reduced ejection fraction of 45.1% (biplane Simpson's method). Ambulatory Holter monitoring exhibited repeated episodes of nonsustained polymorphic ventricular tachycardia. Cardiac magnetic resonance (CMR) imaging 2 days after presentation revealed global left ventricular dysfunction with an ejection fraction of 41%, marked hypokinesia, high T2 values in the apical to mid portion of the anterior wall, and diffuse multifocal patchy late gadolinium enhancement (LGE) (Figure 2).

The patient was admitted to the intensive care unit for the monitoring of hemodynamic and electrical instability and treated with 1 g/kg of intravenous immunoglobulin for two consecutive days as well as 2 mg/kg/day of intravenous methylprednisolone. Ibuprofen use was discontinued after severe myocardial inflammation was identified using CMR imaging. The patient was administered intravenous milrinone and furosemide for a brief period in consideration of the possibility of progressive deterioration of hemodynamic status. Therapy with an angiotensin-converting enzyme inhibitor and beta blocker was initiated after discontinuation of intravenous milrinone.

The troponin I concentration peaked 2 days after presentation (0.689 ng/ml); subsequently, it gradually declined and reached its nadir 9 days after presentation (0.153 ng/ml). Thereafter, it resumed its rising trend, and the patient was administered 1 g/kg of pulsed methylprednisolone therapy for three consecutive days, followed by a planned oral prednisolone taper. CMR imaging 18 days after presentation continued to demonstrate high T2 values in the apical to mid portion of the anterior wall and a slightly decreased extent of diffuse multifocal patchy LGE. A right ventricular endomyocardial biopsy was performed to exclude other etiologies of myocarditis, including giant cell myocarditis. Histological examination of the biopsy specimen revealed focal myocardial degeneration and interstitial edema without significant inflammatory cell infiltration. The patient was discharged 31 days after presentation due to the reduced burden of ventricular tachycardia, improved symptoms, and partial recovery of ventricular function.

Nine days later, the patient was re-hospitalized for *Campylobacter* colitis and treated with intravenous antibiotics, a stress dose of intravenous hydrocortisone, and an intravenous vasopressor during hospitalization. On follow-up 1 month later, a repeat echocardiography revealed left ventricular dilatation with a reduced ejection fraction of 49.1% (biplane Simpson's method), and the troponin I concentration remained above the normal range (0.137 ng/ml). Follow-up CMR imaging was scheduled two months after hospital discharge (Table 1).

**Table 1:** Timeline of case.

Time	Event
October 18, 2021	First dose of BNT162b vaccine
October 20, 2021	Generalized malaise, headache, chest pain, dyspnea on exertion
October 23, 2021	Palpitation, increased intensity of chest pain
October 24, 2021	The patients visited the emergency department of our hospital
	Low voltage in the limb leads on electrocardiogram
	Troponin I level 0.032 ng/ml (normal: 0–0.045 ng/ml)
October 25, 2021	Loss of consciousness for several minutes
October 26, 2021	The patient was admitted to the intensive care unit
	Echocardiography revealed left ventricular dilatation and dysfunction
	Holter monitoring showed nonsustained polymorphic ventricular tachycardia
	1 g/kg IVIG for two consecutive days, 2 mg/kg methylprednisolone, intravenous milrinone
October 27, 2021	CMR imaging findings satisfied with criteria for myocarditis
	ACE inhibitor and beta blocker initiated after discontinuation of milrinone
November 3, 2021	Troponin I level 0.153 ng/ml (nadir)
November 10, 2021	1 g/kg of pulsed methylprednisolone therapy for three consecutive days because of increased cardia
November 12, 2021	CMR imaging showed persistent late gadolinium enhancement
November 18, 2021	Endomyocardial biopsy showed focal myocardial degeneration and interstitial edema
November 25, 2021	Discharge
December 4–7, 2021	Re-hospitalized for *Campylobacter* colitis and treated with intravenous antibiotics, a stress dose of intravenous hydrocortisone, and an intravenous vasopressor
December 24, 2021	Echocardiography revealed sustained left ventricular dysfunction
	Troponin I level 0.137 ng/ml

IVIG, Intravenous immunoglobulin; CMR, Cardiac Magnetic Resonance; ACE, Angiotensin-converting enzyme.

## Discussion

The patient in this report, who developed myocarditis after the first BNT162b vaccine dose, presented with syncope. Since her ventricular function was impaired, and nonsustained ventricular tachycardia was frequent, the patient required electrical and hemodynamic monitoring in the intensive care unit. The patient's cardiac function did not fully recover, with the persistence of elevated cardiac enzymes and residual LGE on CMR imaging.

A large proportion of patients with myocarditis have experienced cardiac arrhythmia at any stage of the disease. The most serious types of arrhythmias have been ventricular tachycardia and ventricular fibrillation. Ventricular arrhythmia has been associated with poor patient outcomes, including the use of mechanical circulatory support and death (19, 20). This arrhythmia potentially manifests as cardiopulmonary arrest and sudden cardiac death. Therefore, current guidelines recommend mandatory close monitoring of cardiovascular status (including heart rhythm) in the early phase in the management of patients with myocarditis (21, 22). Although no deaths have been attributed to arrhythmia in patients with C-VAM, some patients had nonsustained ventricular tachycardia (Table 2) (7–9, 15, 16). Moreover, a case of sudden cardiac death due to C-VAM have been reported (18). Therefore, electrical monitoring is crucial in the management of pediatric patients with C-VAM.

**Table 2:** Case series and retrospective studies of coronavirus disease 2019 vaccination-associated myocarditis and myopericarditis in children.

Study	Truong	Jain	Chua^a^	Das^b^	Schauer^c^	Dionne	Nygaard^d^	Tano^e^	Marshall	Snapiri	Puchalski
Reference	(16)	(9)	(6)	(7)	(13)	(8)	(11)	(15)	(10)	(14)	(12)
Cases, *n*	139	63	33	25	16	15	12	8	7	7	5
Company											
BNT162b2	131	59	33	25	16	15	12	8	7	7	5
mRNA-1273	5	4									
JNJ-78436735	1										
Unknown	2										
Age, y	15.8 (12.1–20.3)	15.6 ± 1.8	15.2 (12.7–17.8)	15 (12–17)	15 (12–17)	15 (12–18)	16 (13–17)	16.7 (15.2–17.9)	17 (14–19)	16.8 (16.2–17.6)	17 (15–17)
Male Sex, *n* (%)	126 (91)	58 (92)	28 (85)	22 (88)	15 (94)	14 (93)	10 (83)	8 (100)	7 (100)	7 (100)	5 (100)
Patients presenting after second vaccination, *n* (%)	128 (92)	62 (98)	27 (82)	22 (88)	16 (100)	14 (93)	6 (50)	7 (88)	7 (100)	6 (86)	2 (40)
Time between symptom onset and last vaccine, *d*	2 (0–22)	2.1 ± 1.3	2 (1–26)	2 (0–20)	3 (2–4)	3 (1–6)	4 (1–39)	2.5 (1–4)	2 (2–4)	2 (1–3)	2 (2–23)
Chest pain, *n* (%)	138 (99)	63 (100)	32 (97)	25 (100)	16 (100)	15 (100)	12 (100)	8 (100)	7 (100)	7 (100)	5 (100)
Fever, *n* (%)	43 (31)	28 (44)	9 (27)	6 (24)	6 (37.5)	10 (67)	N/A	1 (12.5)	5 (71)	1 (14.3)	4 (80)
Elevated troponin level, *n* (%)	139 (100)	63 (100)	32 (97)	25 (100)	16 (100)	15 (100)	12 (100)	8 (100)	7 (100)	7 (100)	5 (100)
Reduced left ventricular ejection fraction, *n* (%)	26 (19)	9 (14)	0	2 (8)	2 (2)	3 (20)	3 (25)	0	1 (14)	0	0
Ventricular tachycardia, *n* (%)	7 (5)	3 (5)	0	3 (12)	N/A	1 (7)	N/A	2 (25)	0	0	0
Complete atrioventricular block, *n* (%)	1 (1)	1 (2)	0	0	N/A	0	N/A	0	0	0	0
Cardiac magnetic resonance, *n*	97	56	32	16	16	15	10	3	7	0	5
Late gadolinum enhancement, *n* (%)	74 (76)	49 (88)	18 (56)	15 (94)	15 (94)	12 (80)	N/A	3 (100)	7 (100)		5 (100)
Hospital stay, *d*	2 (0–10)	3.0 ± 1.4	N/A	3 (2–7)	2 (1–4)	2 (1–5)	4 (3–10)	56.5 (34–95) h	4 (2–6)	5 (3–6)	12 (10–16)
Intensive care unit admission, *n* (%)	26 (19)	27 (43)	0	0	0	0	1 (8)	0	0	4	0
Inotropic/vasoactive support, *n* (%)	2 (1)	0	0	0	0	0	N/A	0	0	0	0
Mechanical circulatory support, *n* (%)	0	0	0	0	0	0	N/A	0	0	0	0
Intravenous immunoglobulin, *n* (%)	30 (22)	17 (27)	0	2 (8)	3 (19)	7 (47)	1 (8)	1 (13)	4 (57)	0	0
Steroid, *n* (%)	30 (22)	15 (24)	0	1 (4)	2 (13)	7 (47)	1 (8)	2 (25)	4 (57)	0	0
Death	0	0	0	0	0	0	0	0	0	0	0

Data are presented as number (%), median (range), or mean ± standard deviation.

N/A; Not applicable.

^a^ Two patients with definite pericarditis were excluded and two patients presented >14 days after vaccination were included.

^b^ Three patients did not require hospitalization.

^c^ This study only included patients with myocarditis following the second dose of the BNT162b2 vaccine.

^d^ Three patients with pericarditis were excluded.

^e^ One patient was diagnosed with perimyocarditis after the first and second dose of the BNT162b2 vaccine, respectively.

Although endomyocardial biopsy (EMB) is the gold standard for the diagnosis of myocarditis, CMR imaging is currently adopted for the confirmation of myocarditis (21). The CMR imaging findings of the patient in this report satisfied the updated Lake Louise criteria, and the patient's condition was consistent with the CDC's definition of confirmed myocarditis. However, the EMB results did not reveal significant inflammatory cell infiltration. These findings possibly resulted from sampling errors associated with the focal distribution of inflammatory infiltrates. The sites of inflammatory infiltrates were sometimes inaccessible to the bioptomes. The false negative rates of EMB were 45% for the left ventricle and 37% for the right ventricle in 38 autopsied hearts with lymphocytic myocarditis (23). The sampling error also occurred due to difference between biopsy sites and involved regions on CMR imaging (24). The biopsy site was usually the right ventricle, while CMR imaging demonstrated predominant left ventricular involvement.

A few case reports and case series showed the histopathologic findings in C-VAM (18, 25–30). The marked inflammatory infiltrates with a predominance of T-cells and macrophages, occasionally admixed with eosinophils, B cells, and plasma cells, and multifocal cardiomyocyte damages were demonstrated in patients with C-VAM (27–30). The autopsied heart with sudden death after COVID-19 vaccination revealed diffuse inflammatory infiltrates predominantly composed of macrophages and neutrophils and the existence of contraction band necrosis (18, 26). However, similar to the findings of the patient in this report, the results of endomyocardial biopsy in patients with C-VAM occasionally demonstrated no inflammatory infiltrates or findings incompatible with classic histopathologic criteria of myocarditis (25, 30–32).

The heart function of the patient in this report was persistently impaired, and LGE was sustained on short-term follow-up CMR imaging. LGE was an independent predictor of mortality and major adverse cardiac events in adult patients with myocarditis (33, 34). The midwall septal pattern of LGE has been associated with late progressive deterioration of left ventricular function (35). Recent studies investigating changes in CMR imaging findings in patients with C-VAM have demonstrated sustained and decreased LGE on follow-up CMR imaging (13, 36, 37). Persistent LGE was observed in a considerable proportion of adult and pediatric patients with myocarditis on follow-up CMR imaging at 3–6 months, even after normalization of inflammatory and cardiac markers (38, 39). Although LGE without edema at 6-month follow-up CMR imaging was associated with a worse outcome in adult patients with acute myocarditis, the clinical significance of LGE and longitudinal changes in heart function in C-VAM require further investigation (40).

## Conclusion

We described an adolescent woman with myocarditis after BNT162b2 mRNA vaccination, who exhibited frequent episodes of nonsustained ventricular tachycardia and persistent left ventricular dysfunction with sustained LGE. Monitoring the electrical and functional cardiac abnormalities in patients with C-VAM is crucial. Further studies focusing on the long-term outcomes and prognosis of patients with C-VAM are warranted.

## Data Availability

The original contributions presented in the study are included in the article, further inquiries can be directed to the corresponding author.

## References

[1] Preston LE Chevinsky JR Kompaniyets L Lavery AM Kimball A Boehmer TK Characteristics and disease severity of US children and adolescents diagnosed with COVID-19. JAMA Netw Open. (2021) 4(4):e215298. 10.1001/jamanetworkopen.2021.5298 33835179 PMC8035649

[2] Son MBF Murray N Friedman K Young CC Newhams MM Feldstein LR Multisystem inflammatory syndrome in children—initial therapy and outcomes. N Engl J Med. (2021) 385(1):23–34. 10.1056/NEJMoa2102605 34133855 PMC8220972

[3] Creech CB Anderson E Berthaud V Yildirim I Atz AM Melendez Baez I Evaluation of mRNA-1273 COVID-19 vaccine in children 6 to 11 years of age. N Engl J Med. (2022) 386(21):2011–23. 10.1056/NEJMoa2203315 35544369 PMC9127699

[4] Walter EB Talaat KR Sabharwal C Gurtman A Lockhart S Paulsen GC Evaluation of the BNT162b2 COVID-19 vaccine in children 5 to 11 years of age. N Engl J Med. (2022) 386(1):35–46. 10.1056/NEJMoa2116298 34752019 PMC8609605

[5] Woodworth KR Moulia D Collins JP Hadler SC Jones JM Reddy SC The advisory committee on immunization practices’ interim recommendation for use of pfizer-BioNTech COVID-19 vaccine in children aged 5–11 years—United States, november 2021. MMWR Morb Mortal Wkly Rep. (2021) 70(45):1579–83. 10.15585/mmwr.mm7045e1 34758012 PMC8580204

[6] Chua GT Kwan MYW Chui CSL Smith RD Cheung EC Tian T Epidemiology of acute myocarditis/pericarditis in Hong Kong adolescents following comirnaty vaccination. Clin Infect Dis. (2022) 75(4):673–81. 10.1093/cid/ciab989 34849657 PMC8767823

[7] Das BB Kohli U Ramachandran P Nguyen HH Greil G Hussain T Myopericarditis after messenger RNA coronavirus disease 2019 vaccination in adolescents 12 to 18 years of age. J Pediatr. (2021) 238:26–32.e1. 10.1016/j.jpeds.2021.07.044 34339728 PMC8321962

[8] Dionne A Sperotto F Chamberlain S Baker AL Powell AJ Prakash A Association of myocarditis with BNT162b2 messenger RNA COVID-19 vaccine in a case series of children. JAMA Cardiol. (2021) 6(12):1446–50. 10.1001/jamacardio.2021.3471 34374740 PMC8356143

[9] Jain SS Steele JM Fonseca B Huang S Shah S Maskatia SA COVID-19 vaccination-associated myocarditis in adolescents. Pediatrics. (2021) 148(5):e2021053427. 10.1542/peds.2021-053427 34389692

[10] Marshall M Ferguson ID Lewis P Jaggi P Gagliardo C Collins JS Symptomatic acute myocarditis in 7 adolescents after pfizer-BioNTech COVID-19 vaccination. Pediatrics. (2021) 148(3):e2021052478. 10.1542/peds.2021-052478 34088762

[11] Nygaard U Holm M Bohnstedt C Chai Q Schmidt LS Hartling UB Population-based incidence of myopericarditis after COVID-19 vaccination in danish adolescents. Pediatr Infect Dis J. (2022) 41(1):e25–e8. 10.1097/INF.0000000000003389 34889875 PMC8658061

[12] Puchalski M Kaminska H Bartoszek M Brzewski M Werner B. COVID-19-vaccination-induced myocarditis in teenagers: case series with further follow-up. Int J Environ Res Public Health. (2022) 19(6):3456. 10.3390/ijerph19063456 35329143 PMC8954790

[13] Schauer J Buddhe S Gulhane A Sagiv E Studer M Colyer J Persistent cardiac magnetic resonance imaging findings in a cohort of adolescents with post-coronavirus disease 2019 mRNA vaccine myopericarditis. J Pediatr. (2022) 245:233–7. 10.1016/j.jpeds.2022.03.032 35351530 PMC8957353

[14] Snapiri O Rosenberg Danziger C Shirman N Weissbach A Lowenthal A Ayalon I Transient cardiac injury in adolescents receiving the BNT162b2 mRNA COVID-19 vaccine. Pediatr Infect Dis J. (2021) 40(10):e360–e3. 10.1097/INF.0000000000003235 34077949 PMC8443419

[15] Tano E San Martin S Girgis S Martinez-Fernandez Y Sanchez Vegas C. Perimyocarditis in adolescents after pfizer-BioNTech COVID-19 vaccine. J Pediatric Infect Dis Soc. (2021) 10(10):962–6. 10.1093/jpids/piab060 34319393 PMC8344528

[16] Truong DT Dionne A Muniz JC McHugh KE Portman MA Lambert LM Clinically suspected myocarditis temporally related to COVID-19 vaccination in adolescents and young adults: suspected myocarditis after COVID-19 vaccination. Circulation. (2022) 145(5):345–56. 10.1161/CIRCULATIONAHA.121.056583 34865500

[17] Abbate A Gavin J Madanchi N Kim C Shah PR Klein K Fulminant myocarditis and systemic hyperinflammation temporally associated with BNT162b2 mRNA COVID-19 vaccination in two patients. Int J Cardiol. (2021) 340:119–21. 10.1016/j.ijcard.2021.08.018 34416319 PMC8372420

[18] Choi S Lee S Seo JW Kim MJ Jeon YH Park JH Myocarditis-induced sudden death after BNT162b2 mRNA COVID-19 vaccination in Korea: case report focusing on histopathological findings. J Korean Med Sci. (2021) 36(40):e286. 10.3346/jkms.2021.36.e286 34664804 PMC8524235

[19] Miyake CY Teele SA Chen L Motonaga KS Dubin AM Balasubramanian S In-hospital arrhythmia development and outcomes in pediatric patients with acute myocarditis. Am J Cardiol. (2014) 113(3):535–40. 10.1016/j.amjcard.2013.10.021 24332245

[20] Othman HF Byrnes J Elsamny E Hamzah M. Impact of ventricular arrhythmias on outcomes in children with myocarditis. Eur J Pediatr. (2020) 179(11):1779–86. 10.1007/s00431-020-03687-4 32447560

[21] Law YM Lal AK Chen S Cihakova D Cooper LT Jr. Deshpande S Diagnosis and management of myocarditis in children: a scientific statement from the American heart association. Circulation. (2021) 144(6):e123–e35. 10.1161/CIR.0000000000001001 34229446

[22] Kociol RD Cooper LT Fang JC Moslehi JJ Pang PS Sabe MA Recognition and initial management of fulminant myocarditis: a scientific statement from the American heart association. Circulation. (2020) 141(6):e69–92. 10.1161/CIR.0000000000000745 31902242

[23] Hauck AJ Kearney DL Edwards WD. Evaluation of postmortem endomyocardial biopsy specimens from 38 patients with lymphocytic myocarditis: implications for role of sampling error. Mayo Clin Proc. (1989) 64(10):1235–45. 10.1016/S0025-6196(12)61286-5 2593714

[24] Chow LH Radio SJ Sears TD McManus BM. Insensitivity of right ventricular endomyocardial biopsy in the diagnosis of myocarditis. J Am Coll Cardiol. (1989) 14(4):915–20. 10.1016/0735-1097(89)90465-8 2794278

[25] Amemiya K Kobayashi T Kataoka Y Iwai T Nakagawa S Morita Y Myocarditis after COVID-19 mRNA vaccination in three young adult males: significance of biopsy in vaccine-associated myocarditis. Pathol Int. (2022) 72(7):385–7. 10.1111/pin.13234 35583173 PMC9347403

[26] Gill JR Tashjian R Duncanson E. Autopsy histopathologic cardiac findings in 2 adolescents following the second COVID-19 vaccine dose. Arch Pathol Lab Med. (2022) 146(8):925–9. 10.5858/arpa.2021-0435-SA 35157759

[27] Kazama S Okumura T Kimura Y Ito R Araki T Mizutani T Biopsy-proven fulminant myocarditis requiring mechanical circulatory support following COVID-19 mRNA vaccination. CJC Open. (2022) 4(5):501–5. 10.1016/j.cjco.2022.02.004 35187464 PMC8842092

[28] Lim Y Kim MC Kim KH Jeong IS Cho YS Choi YD Case report: acute fulminant myocarditis and cardiogenic shock after messenger RNA coronavirus disease 2019 vaccination requiring extracorporeal cardiopulmonary resuscitation. Front Cardiovasc Med. (2021) 8:758996. 10.3389/fcvm.2021.758996 34778411 PMC8586196

[29] Verma AK Lavine KJ Lin CY. Myocarditis after COVID-19 mRNA vaccination. N Engl J Med. (2021) 385(14):1332–4. 10.1056/NEJMc2109975 34407340 PMC8385564

[30] Yamamoto M Tajiri K Ayuzawa S Ieda M. Pathological findings of clinically suspected myocarditis temporally associated with COVID-19 vaccination. Eur J Heart Fail. (2022) 24(6):1132–8. 10.1002/ejhf.2523 35488842 PMC9348161

[31] Larson KF Ammirati E Adler ED Cooper LT Jr. Hong KN Saponara G Myocarditis after BNT162b2 and mRNA-1273 vaccination. Circulation. (2021) 144(6):506–8. 10.1161/CIRCULATIONAHA.121.055913 34133884 PMC8340725

[32] Rosner CM Genovese L Tehrani BN Atkins M Bakhshi H Chaudhri S Myocarditis temporally associated with COVID-19 vaccination. Circulation. (2021) 144(6):502–5. 10.1161/CIRCULATIONAHA.121.055891 34133885 PMC8340723

[33] Grun S Schumm J Greulich S Wagner A Schneider S Bruder O Long-term follow-up of biopsy-proven viral myocarditis: predictors of mortality and incomplete recovery. J Am Coll Cardiol. (2012) 59(18):1604–15. 10.1016/j.jacc.2012.01.007 22365425

[34] Grani C Eichhorn C Biere L Murthy VL Agarwal V Kaneko K Prognostic value of cardiac magnetic resonance tissue characterization in risk stratifying patients with suspected myocarditis. J Am Coll Cardiol. (2017) 70(16):1964–76. 10.1016/j.jacc.2017.08.050 29025553 PMC6506846

[35] Aquaro GD Perfetti M Camastra G Monti L Dellegrottaglie S Moro C Cardiac MR with late gadolinium enhancement in acute myocarditis with preserved systolic function: ITAMY study. J Am Coll Cardiol. (2017) 70(16):1977–87. 10.1016/j.jacc.2017.08.044 29025554

[36] Amir G Rotstein A Razon Y Beyersdorf GB Barak-Corren Y Godfrey ME CMR imaging 6 months after myocarditis associated with the BNT162b2 mRNA COVID-19 vaccine. Pediatr Cardiol. (2022) 43(7):1522–29. 10.1007/s00246-022-02878-0 35320390 PMC8941830

[37] Hadley SM Prakash A Baker AL de Ferranti SD Newburger JW Friedman KG Follow-up cardiac magnetic resonance in children with vaccine-associated myocarditis. Eur J Pediatr. (2022) 181(7):2879–83. 10.1007/s00431-022-04482-z 35482094 PMC9046711

[38] Berg J Kottwitz J Baltensperger N Kissel CK Lovrinovic M Mehra T Cardiac magnetic resonance imaging in myocarditis reveals persistent disease activity despite normalization of cardiac enzymes and inflammatory parameters at 3-month follow-up. Circ Heart Fail. (2017) 10(11):e004262. 10.1161/CIRCHEARTFAILURE.117.004262 29158437

[39] Dubey S Agarwal A Nguyen S Adebo D. Persistence of late gadolinium enhancement on follow-up CMR imaging in children with acute myocarditis. Pediatr Cardiol. (2020) 41(8):1777–82. 10.1007/s00246-020-02445-5 32920654

[40] Aquaro GD Ghebru Habtemicael Y Camastra G Monti L Dellegrottaglie S Moro C Prognostic value of repeating cardiac magnetic resonance in patients with acute myocarditis. J Am Coll Cardiol. (2019) 74(20):2439–48. 10.1016/j.jacc.2019.08.1061 31727281

